# Mortality at one year after transcatheter aortic valve replacement – Relation of age and comorbidities

**DOI:** 10.1016/j.ijcha.2022.101157

**Published:** 2022-11-30

**Authors:** Jarl E. Strange, Emil L. Fosbøl, Caroline Sindet-Pedersen, Eva Havers-Borgersen, Lars Køber, Gunnar H. Gislason, Jonas B. Olesen

**Affiliations:** a Department of Cardiology, Copenhagen University Hospital Herlev and Gentofte, Hellerup, Denmark; b Department of Cardiology, The Heart Center, Copenhagen University Hospital Rigshospitalet, Copenhagen, Denmark; c The Danish Heart Foundation, Copenhagen, Denmark; d Department of Clinical Medicine, Faculty of Health and Sciences, University of Copenhagen, Copenhagen, Denmark

**Keywords:** Transcatheter aortic valve replacement, Mortality, Comorbidities, Age, Prognosis, CI, confidence interval, CKD, Chronic kidney disease, COPD, chronic obstructive pulmonary disease, eGFR, estimated glomerular filtration rate, ICD-10, International classification of Diseases - 10th edition, IQR, interquartile range, TAVR, Transcatheter aortic valve replacement

## Abstract

**Background:**

Of patients undergoing transcatheter aortic valve replacement (TAVR), 80–90 % are at extreme, high, or intermediate risk. Patient selection considering futile outcomes in these groups is difficult as significant comorbidity burden is common. Thus, we examined 1-year mortality after TAVR according to age and comorbidities.

**Methods:**

Between 2008 and 2021 all Danish TAVR-patients were included. From a multivariate Cox-regression model, significant characteristics associated with 1-year all-cause mortality were identified. The study population was divided into four groups according to number of significant comorbidities present at baseline: Low (0 comorbidities), mild (1 comorbidity), moderate (2 comorbidities), and high (3 or more comorbidities). The 1-year risk of all-cause mortality with 95 % confidence intervals (CI) was estimated by each group.

**Results:**

In total, 7,104 patients underwent TAVR. Significant covariates associated with 1-year all-cause mortality were chronic kidney disease, heart failure, chronic obstructive pulmonary disease, peripheral artery disease, and age ≥ 85 years. The four baseline groups comprised low (n = 2,666), mild (n = 2,814), moderate (n = 1,246), and high comorbidity burden (n = 378). The 1-year risk of all-cause mortality was 5.5 % (95 %CI: 4.6–6.4 %) in the low baseline comorbidity burden group. Conversely, the 1-year risk of all-cause mortality was 25.0 % (95 %CI: 20.4–29.3 %) in the high baseline burden group.

**Conclusions:**

In a national sample of TAVR patients, readily available information on age and comorbidities, can be used to identify a high-risk group with 25 % 1-year mortality. This provides physicians and patients with an easy-to-understand view on 1-year prognosis after TAVR and may complement patient selection for improved long-term outcomes.

## 1 Introduction

Avoiding futile outcomes and identifying patients with potential long-term benefits of transcatheter aortic valve replacement (TAVR) is a central part of patient selection. Over time, TAVR has been compared with surgical aortic valve replacement across multiple risk groups [1]. As such, the use of TAVR to treat severe, symptomatic aortic stenosis is increasing [2], [3], [4]. TAVR is now also used more than surgical aortic valve replacement in numerous countries and is expanding to lower risk-groups as well as to younger patients; however, 8–9 out of 10 patients remain extreme-, high-, or intermediate-risk patients [2], [3]. In these groups, patient selection is difficult considering the risk of futile outcomes as significant comorbidity burden and frailty is common.

Continuously modified risk scores exist to discriminate between high- and low-surgical risk patients [5], [6]. However, these scores were developed for valvular surgery and pertain only to the surgical risk – not long-term survival [5], [6]. This is reflected in recent guidelines which underline that patient selection for TAVR vs surgical aortic valve replacement is difficult and that an individual patient-centered approach should be performed including the patient’s treatment preferences [7], [8].

In summary, patients and physicians face a difficult choice balancing the potential increased life-expectancy and reduced symptom burden with TAVR versus the risk of complications or poor prognosis without TAVR. This is particularly important as up to 30 % of patients experienced limited reduced symptom burden or died within 1 year of TAVR despite high procedural success [9].

Consequently, we wanted to stratify patients in a simple manner according to age and comorbidities and examine 1-year mortality by such simple strata. In doing so, we aimed to provide the clinician and the patient with a readily available and easily understandable view on 1-year prognosis.

## 2 Methods

### 2.1 Data collection and definitions of characteristics

This study leveraged data from Danish nationwide registers. A unique personal identification number given to all permanent Danish residents at birth or immigration allowed for crosslinking of information in the following registers: The Danish Civil Registration System [10], The Danish National Patient Register [11], The Danish National Prescription Registry [12], and a database containing blood sample results from 4 out of 5 regions in Denmark, as previously described [13]. The positive predictive value of cardiac diagnoses, procedures, and surgeries is high and appropriate for research [14], [15]. We conducted an observational cohort study in which all patients undergoing first-time TAVR in Denmark between 1 January 2008 and 31 December 2021 were identified.

Baseline characteristics were identified as a hospital contact up to 10 years prior to date of TAVR using International Classification of Diseases − 10th edition (ICD-10) codes (**Table S1** for full list of diagnoses codes). Only hospital admissions or outpatient contacts with a primary or secondary diagnosis of an ICD-10 code were included. Further, a claimed prescription of glucose-lowering drugs within 180 days was used as a proxy for diabetes [16]. Likewise, claimed prescriptions for two or more blood pressure lowering drugs within 180 days was used as a proxy for hypertension [17]. For comedication, claimed prescriptions 180 days prior to TAVR was defined as baseline drug use.

### 2.2 Statistical analyses

Annual procedure volume per TAVR-center is presented. Baseline characteristics are presented with numbers and percentages for categorical values and median and interquartile ranges (IQR) for numerical values. Further, baseline characteristics for each significant covariate associated with 1-year all-cause mortality is presented in **Table S2.**

#### 2.2.1 Stratification of study population and outcome

Significant characteristics of patients associated with 1-year risk of all-cause death were identified from a multivariate Cox regression model. The following variables were entered into the model based on a clinical assessment of relevant factors for poor long-term survival: Sex, age (categorial: <85 years, ≥85 years), history of stroke, myocardial infarction, heart failure, diabetes, peripheral artery disease, chronic obstructive pulmonary disease (COPD), chronic kidney disease (CKD), and calendar year group of procedure. Based on covariates significantly associated with 1-year mortality (Figure S1 for estimates), the cohort was then divided according to the number of significant comorbidities (including age ≥ 85 years) present at baseline; 0 comorbidities, 1 comorbidity, 2 comorbidities, and 3 or more comorbidities to reflect low, mild, moderate, and high baseline comorbidity burden groups, respectively. However, calendar year of procedure was used only as an adjustment variable – not included in the score. This was done to account for improvements in procedural technique and patient selection not otherwise captured by covariates already included in the model The 1-year risk of all-cause death for each group was non-parametrically estimated and compared using the log-rank test. We aimed to describe mortality rates by these baseline groups. In doing so, patients were followed from date of TAVR procedure until death, emigration, one year of follow-up, or 31 December 2021, whichever came first.

#### 2.2.2 Sensitivity and supplementary analyses

In a sensitivity analysis, we evaluated if the inclusion of patients with an available blood sample of hemoglobin, creatinine, and albumin would refine the identification of patients with poor prognosis. From the baseline creatinine level, the estimated glomerular filtration rate (eGFR) was estimated using the CKD-EPI formula [18]. The same approach was used as in the main analysis, however, the Cox regression model was adjusted for sex, age (categorial: <85 years, ≥85 years), history of stroke, myocardial infarction, heart failure, diabetes, peripheral artery disease, COPD, hemoglobin (categorial: ≤6 mmol/L, >6 mmol/L), eGFR (categorial: ≤29 ml/min/1.73 m^2^, ≥30 ml/min/1.73 m^2^), albumin (categorial: ≤29 g/L, ≥30 g/L).

We performed several supplementary analyses:

i) To investigate the association between age and mortality alone, we stratified the population into age quintiles and estimated the 1-year risk of all-cause death within each quintile with the aim of providing insights into the impact of age compared with comorbidities on mortality.
ii) We evaluated the 1-year risk of death individually for each significant comorbidity i.e. heart failure vs. no heart failure. This was done to investigate differences in the importance of each comorbidity.
iii) As CKD was significantly associated with 1-year mortality in both the main analysis using diagnoses codes and the sensitivity analyses including biomarkers, we stratified patients from the sensitivity analyses into four eGFR groups: eGFR ≥ 90, eGFR 60–89, eGFR 30–59, and eGFR < 30 and estimated the 1-year risk of all-cause death within each group. This was done to investigate levels of eGFR on the risk of death.

Data, statistical analyses, and figures were managed, performed, and created in R [19].

### 2.3 Ethics

The present study was approved by the data responsible institution, Capital Region. We refer to approval number P-2019–191. In Denmark, retrospective registry-based cohort studies do not require further approval from the Research Ethics Committee System.

## 3 Results

### 3.1 Population characteristics

During the study period (2008–2021), 7,104 patients underwent first-time TAVR in Denmark. Significant baseline characteristics associated with 1-year mortality were the five covariates: CKD, COPD, heart failure, peripheral artery disease, and age ≥ 85 years (Figure S1). Patients were divided into 4 groups according to baseline comorbidity burden: low baseline comorbidity burden (none of the five risk factors, n = 2,666), mild baseline comorbidity burden (one of the five risk factors, n = 2,814), moderate comorbidity burden (two of the five risk factors, n = 1,246), and high baseline comorbidity burden (at least three of the five risk factors, n = 378). Procedure volume increased over time with three out of four centers performing>50 first-time TAVR procedures annually from 2011 **(**Figure S2**).** All centers performed>50 first-time TAVR procedures from 2016.

The baseline characteristics of patients within each group are shown in Table 1. Patients in the low baseline comorbidity burden group were the youngest (median age 80 IQR [75–82] years). Contrary, patients in the high baseline comorbidity burden group were the oldest (median age 85 IQR [79–87] years). A temporal trend towards treating lower comorbidity burden patients was observed; in 2008–2010 the high baseline comorbidity burden group comprised 35/393 (8.9 %) patients compared with 65/1,970 (3.2 %) in 2020–2021. However, the absolute number of high baseline comorbidity burden patients increased over time.

**Table 1:** Characteristics according to baseline groups.

**Baseline comorbidity burden**	**Low**	**Mild**	**Moderate**	**High**	**Total**
	**0**	**1**	**2**	**3 or more**	
No.	2,666	2,814	1,246	378	7,104
Male (%)	1,442 (54.1)	1,496 (53.2)	743 (59.6)	258 (68.3)	3,939 (55.4)
Age (years), median [IQR]	80 [75–82]	83 [78–87]	85 [78–87]	85 [79–87]	81 [77–85]
Year group*					
2008–2010	108 (27.5)	151 (38.4)	99 (25.2)	35 (8.9)	393 (1 0 0)
2011–2013	238 (27.7)	374 (43.5)	179 (20.8)	69 (8.0)	860 (1 0 0)
2014–2016	500 (32.9)	606 (39.9)	310 (20.4)	103 (6.8)	1,519 (1 0 0)
2017–2019	923 (39.1)	939 (39.8)	394 (16.7)	106 (4.5)	2,362 (1 0 0)
2020–2021	897 (45.5)	744 (37.8)	264 (13.4)	65 (3.3)	1,970 (1 0 0)
**Comorbidities, No. (%)**					
Stroke/systemic embolism	325 (12.2)	378 (13.4)	186 (14.9)	68 (18.0)	957 (13.5)
Myocardial infarction	222 (8.3)	308 (10.9)	202 (16.2)	95 (25.1)	827 (11.6)
Ischemic heart disease	1,014 (38.0)	1,254 (44.6)	674 (54.1)	263 (69.6)	3,205 (45.1)
Heart failure	0 (0)	779 (27.7)	856 (68.7)	337 (89.2)	1,972 (27.8)
Peripheral artery disease	0 (0)	271 (9.6)	347 (27.8)	211 (55.8)	829 (11.7)
Previous PCI	508 (19.1)	604 (21.5)	368 (29.5)	136 (36.0)	1,616 (22.7)
Previous CABG	106 (4.0)	107 (3.8)	66 (5.3)	34 (9.0)	313 (4.4)
Atrial fibrillation	728 (27.3)	984 (35.0)	532 (42.7)	201 (53.2)	2,445 (34.4)
Diabetes	490 (18.4)	506 (18.0)	252 (20.2)	103 (27.2)	1,351 (19.0)
COPD	0 (0)	359 (12.8)	367 (29.5)	235 (62.2)	961 (13.5)
Chronic kidney disease	0 (0)	217 (7.7)	280 (22.5)	202 (53.4)	699 (9.8)
Hemoglobin ≤ 6 mmol/L	1,727 (91.0)	1,682 (86.7)	708 (82.8)	206 (84.8)	4,323 (87.6)
Missing	769 (28.8)	875 (31.1)	391 (31.4)	135 (35.7)	2,170 (30.5)
eGFR < 30 ml/min/1,73 m^2^	1,883 (98.5)	1,857 (95.1)	732 (85.3)	180 (74.1)	4,652 (93.7)
Missing	755 (28.3)	862 (30.6)	388 (31.1)	135 (35.7)	2,140 (30.1)
Albumin < 30 g/L	1,423 (90.5)	1,436 (85.9)	629 (83.9)	177 (80.1)	3,665 (87.0)
Missing	1,094 (41.0)	1,142 (40.6)	496 (39.8)	157 (41.5)	2,889 (40.7)

Abbreviations: CABG: Coronary artery bypass graft, COPD: Chronic obstructive pulmonary disease, eGFR: Estimated glomerular filtration rate, IQR: Interquartile range, PCI: Percutaneous coronary intervention.

* Percentages represent row percentages.

### 3.2 Mortality according to age and comorbidity burden

**Fig. 1:**
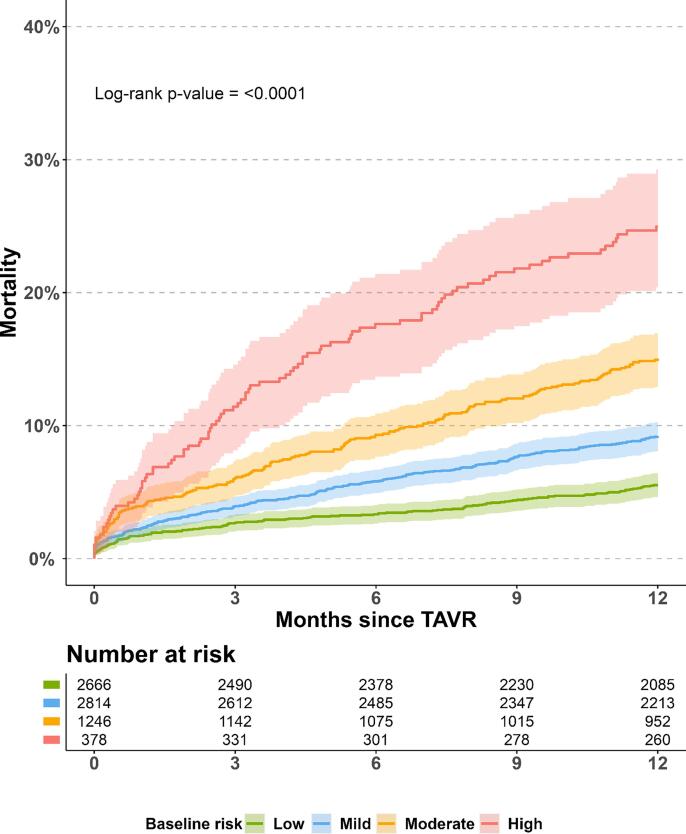
**Title:** Absolute risk of 1-year all-cause mortality. **Legend:** The absolute risk of all-cause mortality for each burden group after TAVR. Numbers beneath plot represents patients at risk 0, 3, 6, 9, and 12 months after TAVR, respectively. Colored areas represent 95 % confidence intervals.

The unadjusted 1-year risk of all-cause mortality for all patients was 9.7 % (95 % confidence interval (CI): 9.0 % to 10.5 %) **(**Figure S3**).** Fig. 1 illustrates the 1-year absolute risk of death for each burden group. The risk of death increased with increasing burden group. Specifically, patients in the low baseline risk group had the lowest risk of death with a 1-year risk of all-cause death of 5.5 % (95 %CI: 4.6 % to 6.4 %). Conversely, patients in the high baseline risk group had the highest risk of death with a 1-year risk of all-cause death of 25.0 % (95 %CI: 20.4 % to 29.3 %).

When analyzing mortality according to each comorbidity factor **(**Figure S4**)**, only history of CKD conferred a point estimate of>20 % 1-year risk of death (20.2 % [95 % CI: 17.1 % to 23.2 %]). Stratifying patients according to age into two groups did not confer significant differences in risk of death and the CI overlapped. Further, when stratifying patients into quintiles based on age groups, the 1-year risk of death increased gradually up to 12.1 % (95 % CI: 10.2 % to 13.9 %) in the oldest age quintile: 87–100 years **(**Figure S5**)**.

### 3.3 Sensitivity analysis with biomarkers

**Fig. 2:**
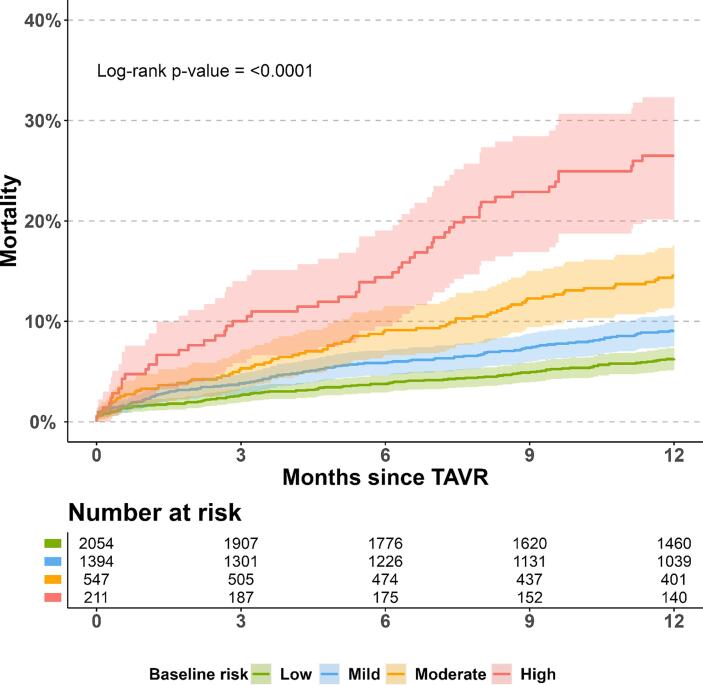
**Title:** Absolute risk of 1-year all-cause mortality with biomarkers. **Legend:** The absolute risk of all-cause mortality for each burden group after TAVR in a population with biomarkers. Numbers beneath plot represents patients at risk 0, 3, 6, 9, and 12 months after TAVR, respectively. Colored areas represent 95 % confidence intervals. The included biomarkers were hemoglobin, estimated glomerular filtration rate, and albumin.

The sensitivity analysis comprised 4,206 TAVR patients with an available sample of hemoglobin, eGFR, and albumin. In the Cox model, heart failure, COPD, and the three biomarkers (hemoglobin ≤ 6 mmol/L, eGFR < 30 ml/min/1.73 m^2^, albumin < 30 g/L) were all significantly associated with an increased hazard ratio of death **(**Figure S6**).** As with the main analysis, the 1-year risk of all-cause mortality increased with increasing baseline comorbidity burden group **(**Fig. 2**).** When stratifying patients solely according to eGFR, the 1-year absolute risk of all-cause death was below 15 % for all groups except for eGFR < 30, which was associated with marked increased risk of death of 21.3 % (95 %CI: 16.3 % to 25.9 %) **(**Figure S7**).**

## 4 Discussion

In this nationwide, observational cohort study, investigating the 1-year risk of all-cause death as a function of baseline age and comorbidity burden, the main findings can be summarized as: i) The proportion of high baseline comorbidity burden patients decreased over time, however, the absolute number of patients in this group increased. ii) When dividing patients according to age and comorbidity burden, a 4-fold increased risk of death was found between high baseline comorbidity burden patients and low baseline comorbidity burden patients. iii) Patients with a one in four risk of death within one year can be identified from few clinical risk factors.

### 4.1 Long-term survival after TAVR

Existing models for patient selection have limited implications for identifying TAVR patients at high risk of 1-year mortality [20]. In this study, we found that a simple approach based on age and simple comorbidity burden was able to identify a subgroup of patients associated with a 25 % risk of death within one year of TAVR. This provides physicians and patients with a readily available tool to discuss prognosis after TAVR.

Other studies have evaluated 1-year mortality in patients undergoing TAVR [21], [22]. Hermiller et al. found that home oxygen use, low albumin, falls in past 6 months, STS PROM score > 7 %, and severe Charlson score predicted 1-year mortality [22]. This study found an overall mortality that was higher (22.8 %) compared to the present study most likely due to inclusion of only high-risk and extreme-risk patients. They found a subset of patients with 36.6 % 1-year risk of death, however, as stated by the authors, the applicability of the results to everyday clinical practice is limited as the predictors included in the study are time consuming to gather and not routinely collected [22]. Kiani et al. found 1-year mortality rates ranging from around 7 % to 27 % depending on the number of frailty components (low albumin, anemia, and slow walking speed) present at time of TAVR. In our sensitivity analysis including blood samples, we also found that low albumin and low hemoglobin was associated with an increased risk of 1-year all-cause death. Laboratory work is routinely collected in clinical practice before patients undergo TAVR and might improve patient selection. Further, albumin and hemoglobin levels are increasingly recognized as markers of frailty and has been associated with an increased risk of death in previous studies [21], [22], [23], [24], [25].

### 4.2 Comorbidities and age in relation to mortality

We found that CKD whether based on a diagnosis code or baseline eGFR ≤ 30 was associated with the greatest increase in the 1-year risk of death. Patients with CKD represents an important subgroup, as patients with severe CKD or dialysis were excluded or underrepresented in all landmark TAVR trials [1]. By contrast, CKD is prevalent in TAVR patients in real world cohorts [26], [27], [28], [29]. In our analysis using only diagnosis codes, CKD was associated with a > 20 % 1-year risk of death. However, this estimate may be higher than what is attributable to CKD alone as residual comorbidity burden was highest for patients with CKD compared to patients with the other significant covariates **(Table S2).** Nevertheless, when restricting the analysis according to eGFR groups, the 1-year risk of death increased with reduced baseline kidney function as expected, however, there was a marked increase in risk of death particularly for patients with eGFR ≤ 30 consistent with previous findings [26], [27], [28], [30]. The most prevalent comorbidity in the score was heart failure. Heart failure is common in TAVR patients [31]. Further, reduced ejection fraction has been associated with an increased risk of short- and long-term mortality despite TAVR improving ejection fraction in low ejection fraction patients [32].

Interestingly, stratifying patients according to age > 85 and 85 years and younger, the survival curves overlapped with only a limited difference in the 1-year mortality point estimates. Further, in our supplementary analysis stratifying patients into age quintiles, the survival curves once again overlapped with only the greatest quintile, age 87–100 years, having a relatively higher point estimate compared to other age groups. Importantly, the mortality in this age group was still lower compared to CKD, heart failure, peripheral artery disease, and COPD. The results indicate a limited ability to discriminate patients based on chronological age alone most likely explained by selection and healthy survivor bias consistent with other findings [33], [34], [35], [36].

## 5 Strengths and limitations

This study leveraged data from Danish nationwide administrative registers. In doing so, all patients undergoing TAVR in Denmark were included thereby minimizing selection bias. Further the data sources in combination with the unique personal identification number allowed for almost complete follow-up for all patients enrolled except for administrative censoring (end of study period). In Denmark, only four high-volume centers perform TAVR through a tax-funded health care system ensuring universal, free access to TAVR minimizing selection bias across socioeconomic groups. Further, high-volume centers with on-site cardiac surgeons facilitates optimal outcomes of TAVR procedures [37].

Some limitations apply: This was not a prospective study. Information on procedural characteristics such as type of bioprosthetic valve and paravalvular leakage are lacking. Important unmeasured confounders were not available in the registers (e.g. smoking habits and frailty indices). Using ICD-10 codes limits the model discrimination i.e. degree of COPD and types of heart failure could not be incorporated in the model. For the sensitivity analysis including laboratory works, not all patients had available information. Changes in kidney function over time were not included. We were not able to investigate mortality among patients who did not undergo TAVR which could have provided valuable information for comparison.

### 5.1 Conclusions

In a retrospective nationwide cohort study national sample of patients undergoing TAVR, we showed that readily available information on age and medical history could easily identify a high-risk patient group with 25 % mortality by 1-year. This may provide physicians and patients with a readily available view on 1-year prognosis after TAVR. Our results suggest that TAVR in patients with chronic kidney disease should be thoroughly reconsidered, but more studies are needed to guide patient selection to secure best patient-level but also society-economic outcomes.

## Declaration of Competing Interest

The authors declare that they have no known competing financial interests or personal relationships that could have appeared to influence the work reported in this paper.
