# Supplementary material for: Mortality at one year after transcatheter aortic valve replacement – Relation of age and comorbidities
Source: Int J Cardiol Heart Vasc. 2022 Nov 30;43:101157. doi: 10.1016/j.ijcha.2022.101157 (PMC9718958; doi:10.1016/j.ijcha.2022.101157)

**Supplemental material**

***Mortality at one year after transcatheter aortic valve replacement – importance of age and comorbidities***

**P.2……………….………………….. List of ICD10-, ATC-, NCSP-, and NPU-codes (Table S1)**

**P.5……………….………….. Baseline characteristics for each significant covariate (Table S2)**

**P.6…………………..……………………………………………...Supplementary Figure legends**

**P.9…………………..……………………………………...…... Cox regression model (Figure S1)**

**P.10……………...…………………..…………………...Procedure volume over time (Figure S2)**

**P.11……….………….………... Overall absolute risk of 1-year all-cause mortality (Figure S3)**

**P.12……..….. Absolute risk of 1-year all-cause mortality for significant covariates (Figure S4)**

**P.13………………..…. Absolute risk of 1-year all-cause mortality for age quintiles (Figure S5)**

**P.14…………………………..…..……..…... Cox regression model with biomarkers (Figure S6)**

**P.15……………….. Absolute risk of 1-year all-cause mortality stratified for eGFR (Figure S7)**

| Supplementary Table 1: List of ICD10-, ATC-, NCSP-, and NPU-codes | | |
| --- | --- | --- |
| Population |  |  |
| Transcatheter Aortic Valve Replacement | Defined from surgical procedures | **NCSP:** KFMD11, KFMD12, KFMD14 |
| Comorbidities |  |  |
| Percutaneous coronary intervention | Defined from surgical procedures | **NCSP:** KFNG00, KFNG02, KFNG05, KFNG10, KFNG12, KFNG96 |
| Coronary artery bypass grafting | Defined from surgical procedures | **NCSP:** KNFA, KFNB, KFNC, KFND, KFNE |
| Ischemic stroke/systemic embolism | Defined from diagnoses codes | **ICD10:** I63, I64, I74, G458, G459 |
| Myocardial infarction | Defined from diagnoses codes | **ICD10:** I21-I22 |
| Ischemic heart disease | Defined from diagnoses codes | **ICD10:** I20-I25 |
| Heart failure | Defined from diagnoses codes | **ICD10**: I110, I130, I132, I420, I426, I427, I428, I429, I50 |
| Atrial fibrillation | Defined from diagnoses codes | **ICD10**: I48 |
| Peripheral artery disease | Defined from diagnoses codes or surgical procedures | **ICD10**: I70, I74, I739  **NCSP:** KPDE, KPDF, KPDG, KPDH, KPDN, KPDP, KPDQ, KPDR, KPDU, KPEE, KPEF, KPEG, KPEH, KPEN, KPEP, KPEQ, KPER, KPEU |
| Hypertension | Defined from diagnoses codes or treatment with 2 or more antihypertensive drugs: adrenergic α-antagonists, non-loop diuretics, vasodilators, β-blockers, calcium channel blockers, renin-angiotensin system inhibitors. | **ICD10**: I10-15 **ATC**: C02A, C02B, C02C, C02L, C03A, C03B, C03D, C03E, C03X, C07B, C07C, C07D, C08G, C02DA, C09BA, C09DA, C02DB, C02DD, C02DG, C07A, C07B, C07C, C07D, C07F, C08, C09BB, C09DB, C09AA, C09BA, C09BB, C09CA, C09DA, C09DB, C09XA02, C09XA52, C03C, C03EB |
| Diabetes mellitus | Defined from glucose-lowering medication | **ATC**: A10 |
| Chronic kidney disease | Defined from diagnoses codes | **ICD10:** E102, E112, E132, E142, I120, N02-N08, N11, N12, N14, N18, N19, N26, N158, N159, N160, N162, N163, N164, N168, N391, Q612, Q613, Q615, Q619, Z940, Z992 |
| Chronic obstructive pulmonary disease | Defined from diagnoses codes | **ICD10**: J44 |
| Pharmacotherapy |  |  |
| Adenosine-phosphate receptor antagonists | Defined from ATC codes | **ATC**: B01AC04, B01AC22, B01AC24 |
| Aspirin | Defined from ATC codes | **ATC**: B01AC06 N02BA01 |
| Non-steroidal-anti-inflammatory-drugs | Defined from ATC codes | **ATC**: M01A, except M01AX05 |
| Beta-blockers | Defined from ATC codes | **ATC**: C07A, C07B, C07C, C07D, C07F |
| Calcium channel blockers | Defined from ATC codes | **ATC**: C07F, C08, C09BB, C09DB |
| Renin-angiotensin system inhibitors | Defined from ATC codes | **ATC**: C09AA, C09BA, C09BB, C09CA, C09DA, C09DB, C09XA02, C09XA52 |
| Loop diuretics | Defined from ATC codes | **ATC**: C03C C03EB |
| Blood sample values |  |  |
| Haemoglobin mmol/L | Defined from NPU code | **NPU:** 02319 |
| Creatinine µmol/L | Defined from NPU codes | **NPU:** 04998, 18016, 17559 |
| Albumin g/L | Defined from NPU codes | **NPU:** 19673, 01132 |
|  |  |  |

| **Supplementary Table 2:** Baseline characteristics for each significant covariate | | | | | | | | | | | | | | |
| --- | --- | --- | --- | --- | --- | --- | --- | --- | --- | --- | --- | --- | --- | --- |
| Characteristics | No CKD (n=6,405) | CKD (n=699) | Characteristics | No HF (n=5,132) | HF (n=1,972) | Characteristics | No COPD (n=6,143) | COPD (n=961) | Characteristics | No PAD (n=6,275) | PAD (n=829) | Characteristics | Age <85 (n=5,063) | Age ≥85 (n=2,041) |
| Male (%) | 3.458 (54.0) | 481 (68.8) | Male (%) | 2,669 (52.0) | 1,270 (64.4) | Male (%) | 3,402 (55.4) | 537 (55.9) | Male (%) | 3,409 (54.3) | 530 (63.9) | Male (%) | 2,957 (58.4) | 982 (48.1) |
| Age (years), median [IQR] | 82 [77–85] | 79 [75–84] | Age (years), median [IQR] | 81 [77–85] | 81 [76–85] | Age (years), median [IQR] | 82 [77–85] | 80 [75–84] | Age (years), median [IQR] | 82 [77–85] | 80 [76–84] | Age (years), median [IQR] | 79 [75–82] | 87 [86–89] |
| Age ≥85 | 1,901 (29.7) | 140 (20.0) | Age ≥85 | 1,461 (28.5) | 580 (29.4) | Age ≥85 | 1,833 (29.8) | 208 (21.6) | Age ≥85 | 1,862 (29.7) | 179 (21.6) | Heart failure | 1,392 (27.5) | 580 (28.4) |
| Heart failure | 1,657 (25.9) | 315 (45.1) | Heart failure | 506 (9.9) | 323 (16.4) | Heart failure | 1,602 (26.1) | 370 (38.5) | Heart failure | 1,649 (26.3) | 323 (39.0) | Peripheral artery disease | 650 (12.8) | 179 (8.8) |
| Peripheral artery disease | 690 (10.8) | 139 (19.9) | Chronic kidney disease | 384 (7.5) | 315 (16.0) | Peripheral artery disease | 652 (10.6) | 177 (18.4) | Chronic kidney disease | 560 (8.9) | 139 (16.8) | Chronic kidney disease | 559 (11.0) | 140 (6.9) |
| COPD | 824 (12.9) | 137 (19.6) | COPD | 591 (11.5) | 370 (18.8) | Chronic kidney disease | 562 (9.1) | 137 (14.3) | COPD | 784 (12.5) | 177 (21.4) | COPD | 753 (14.9) | 208 (10.2) |
| Stroke/systemic embolism | 845 (13.2) | 112 (16.0) | Stroke/systemic embolism | 680 (13.3) | 277 (14.0) | Stroke/systemic embolism | 827 (13.5) | 130 (13.5) | Stroke/systemic embolism | 775 (12.4) | 182 (22.0) | Stroke/systemic embolism | 695 (13.7) | 262 (12.8) |
| Myocardial infarction | 701 (10.9) | 126 (18.0) | Myocardial infarction | 446 (8.7) | 381 (19.3) | Myocardial infarction | 700 (11.4) | 127 (13.2) | Myocardial infarction | 656 (10.5) | 171 (20.6) | Myocardial infarction | 614 (12.1) | 213 (10.4) |
| Ischemic heart disease | 2,813 (43.9) | 392 (56.1) | Ischemic heart disease | 2,082 (40.6) | 1,123 (56.9) | Ischemic heart disease | 2,717 (44.2) | 488 (50.8) | Ischemic heart disease | 2,657 (42.3) | 548 (66.1) | Ischemic heart disease | 2,323 (45.9) | 882 (43.2) |
| Previous PCI | 1,416 (22.1) | 200 (28.6) | Previous PCI | 1,045 (20.4) | 571 (29.0) | Previous PCI | 1,375 (22.4) | 241 (25.1) | Previous PCI | 1,319 (21.0) | 297 (35.8) | Previous PCI | 1,152 (22.8) | 464 (22.7) |
| Previous CABG | 262 (4.1) | 51 (7.3) | Previous CABG | 176 (3.4) | 137 (6.9) | Previous CABG | 274 (4.5) | 39 (4.1) | Previous CABG | 233 (3.7) | 80 (9.7) | Previous CABG | 272 (5.4) | 41 (2.0) |
| Atrial fibrillation | 2,139 (33.4) | 306 (43.8) | Atrial fibrillation | 1,514 (29.5) | 931 (47.2) | Atrial fibrillation | 2,059 (33.5) | 386 (40.2) | Atrial fibrillation | 2,149 (34.2) | 296 (35.7) | Atrial fibrillation | 1,688 (33.3) | 757 (37.1) |
| Diabetes | 1,111 (17.3) | 240 (34.3) | Diabetes | 910 (17.7) | 441 (22.4) | Diabetes | 1,139 (18.5) | 212 (22.1) | Diabetes | 1,140 (18.2) | 211 (25.5) | Diabetes | 1,116 (22.0) | 235 (11.5) |
| Year group |  |  | Year group |  |  | Year group |  |  | Year group |  |  | Year group |  |  |
| 2008-2010 | 346 (5.4) | 47 (6.7) | 2008-2010 | 235 (4.6) | 158 (8.0) | 2008-2010 | 331 (5.4) | 62 (6.5) | 2008-2010 | 335 (5.3) | 58 (7.0) | 2008-2010 | 260 (5.1) | 133 (6.5) |
| 2011-2013 | 766 (12.0) | 94 (13.4) | 2011-2013 | 556 (10.8) | 304 (15.4) | 2011-2013 | 715 (11.6) | 145 (15.1) | 2011-2013 | 729 (11.6) | 131 (15.8) | 2011-2013 | 586 (11.6) | 274 (13.4) |
| 2014-2016 | 1,342 (21.0) | 177 (25.3) | 2014-2016 | 1,042 (20.3) | 477 (24.2) | 2014-2016 | 1,287 (21.0) | 232 (24.1) | 2014-2016 | 1,331 (21.2) | 188 (22.7) | 2014-2016 | 1,042 (20.6) | 477 (23.4) |
| 2017-2019 | 2,142 (33.4) | 220 (31.5) | 2017-2019 | 1,740 (33.9) | 622 (31.5) | 2017-2019 | 2,051 (33.4) | 311 (32.4) | 2017-2019 | 2,116 (33.7) | 246 (29.7) | 2017-2019 | 1,692 (33.4) | 670 (32.8) |
| 2020-2021 | 1,809 (28.2) | 161 (23.0) | 2020-2021 | 1,559 (30.4) | 411 (20.8) | 2020-2021 | 1,759 (28.6) | 211 (22.0) | 2020-2021 | 1,764 (28.1) | 206 (24.8) | 2020-2021 | 1,483 (29.3) | 487 (23.9) |

CABG: Coronary artery bypass grafting. CKD: Chronic kidney disease. COPD: Chronic obstructive pulmonary disease. HF: Heart failure. IQR: Interquartile range. PAD: Peripheral artery disease. PCI: Percutaneous coronary intervention.

**Supplementary Figure legends**

**Supplementary Figure 1:**

**Title:** Cox regression model

**Legend:** Estimates of covariates from a Cox regression model of 1-year all-cause mortality. Abbreviations: CI: Confidence interval. COPD: Chronic obstructive pulmonary disease. PAD: Peripheral artery disease.

*Year group 2008-2010 as reference.

**Supplementary Figure 2:**

**Title:** Procedure volume over time.

**Legend:** Annual procedure volume per center. In Denmark, four centers perform TAVR, and each bar represents an individual TAVR center. Horizontal dashed line represents 50 procedures. Abbreviations: TAVR: Transcatheter aortic valve replacement.

**Supplementary Figure 3:**

**Title:** Overall absolute risk of 1-year all-cause mortality.

**Legend:** The overall absolute risk of all-cause mortality for all patients undergoing TAVR Numbers beneath plot represents patients at risk 0, 3, 6, 9, and 12 months after TAVR, respectively. Colored areas represent 95% confidence intervals. Abbreviations: TAVR: Transcatheter aortic valve replacement.

**Supplementary Figure 4:**

**Title:** Absolute risk of 1-year all-cause mortality for significant covariates.

**Legend:** The absolute risk of all-cause mortality for each individual significant covariate of the Cox-regression model. Panel A: Chronic kidney disease; Panel B: Heart failure. Panel C: Chronic obstructive pulmonary disease; Panel D: Peripheral artery disease; Panel E: Age groups; Numbers beneath plot represents patients at risk 0, 3, 6, 9, and 12 months after TAVR, respectively. Colored areas represent 95% confidence intervals. Abbreviations: CKD: Chronic kidney disease. COPD: Chronic obstructive pulmonary disease. HF: Heart failure. PAD: Peripheral artery disease. TAVR: Transcatheter aortic valve replacement.

**Supplementary Figure 5:**

**Title:** Absolute risk of 1-year all-cause mortality for age quintiles.

**Legend:** The absolute risk of all-cause mortality for TAVR patients stratified into quintiles based on age. Numbers beneath plot represents patients at risk 0, 3, 6, 9, and 12 months after TAVR, respectively. Colored areas represent 95% confidence intervals. Abbreviations: TAVR: Transcatheter aortic valve replacement.

**Supplementary Figure 6:**

**Title:** Cox regression model with biomarkers.

**Legend:** Estimates of covariates from a Cox regression model of 1-year all-cause mortality. Abbreviations: CI: Confidence interval. COPD: Chronic obstructive pulmonary disease. PAD: Peripheral artery disease.

*Year group 2008-2010 as reference.

**Supplementary Figure 7:**

**Title:** Absolute risk of 1-year all-cause mortality stratified for eGFR.

**Legend:** The absolute risk of all-cause mortality for TAVR patients stratified for eGFR which was estimated with the CKD-EPI formula. Numbers beneath plot represents patients at risk 0, 3, 6, 9, and 12 months after TAVR, respectively. Colored areas represent 95% confidence intervals. Abbreviations: eGFR: Estimated glomerular filtration rate. TAVR: Transcatheter aortic valve replacement.

**Supplementary Figure 1**


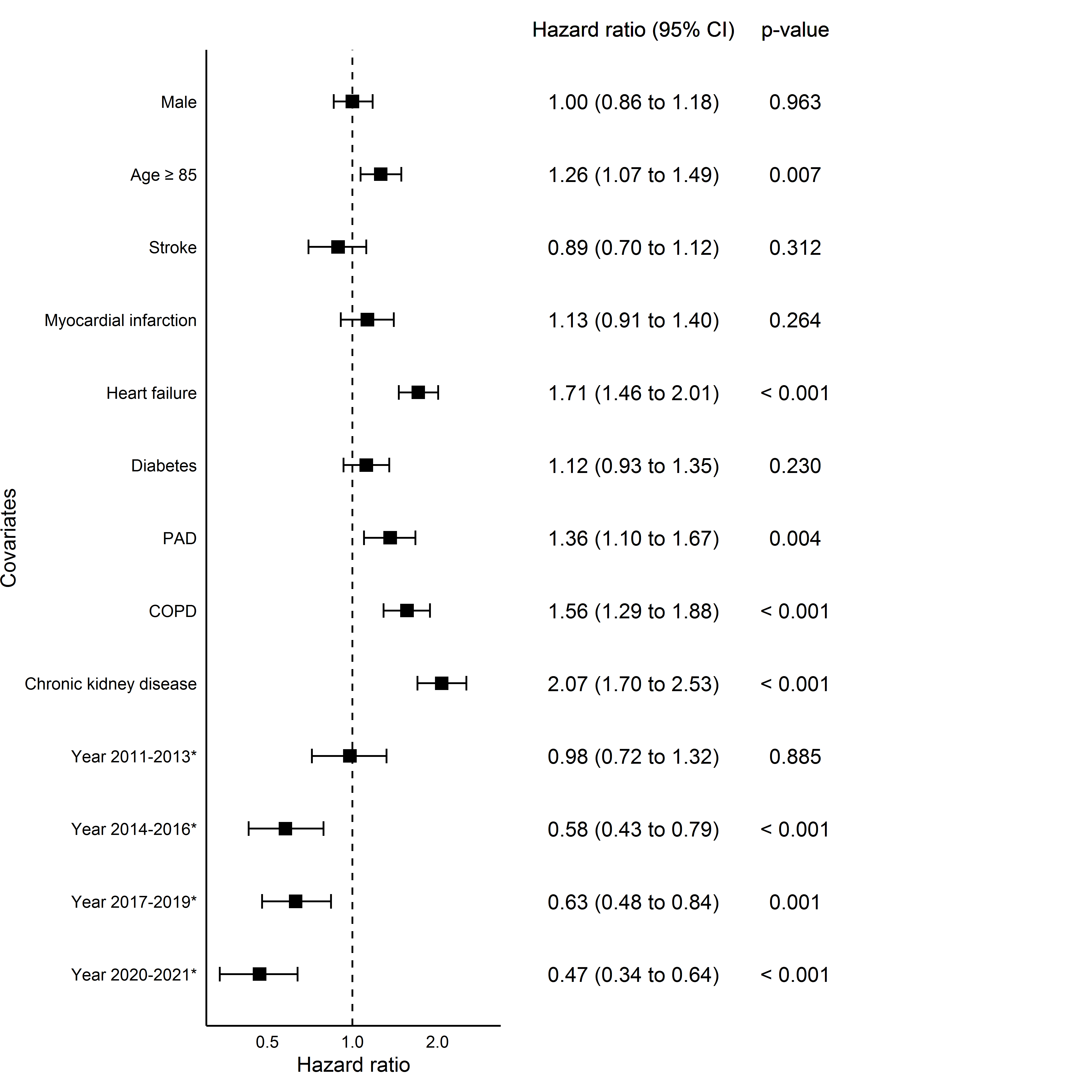


**Supplementary Figure 2**


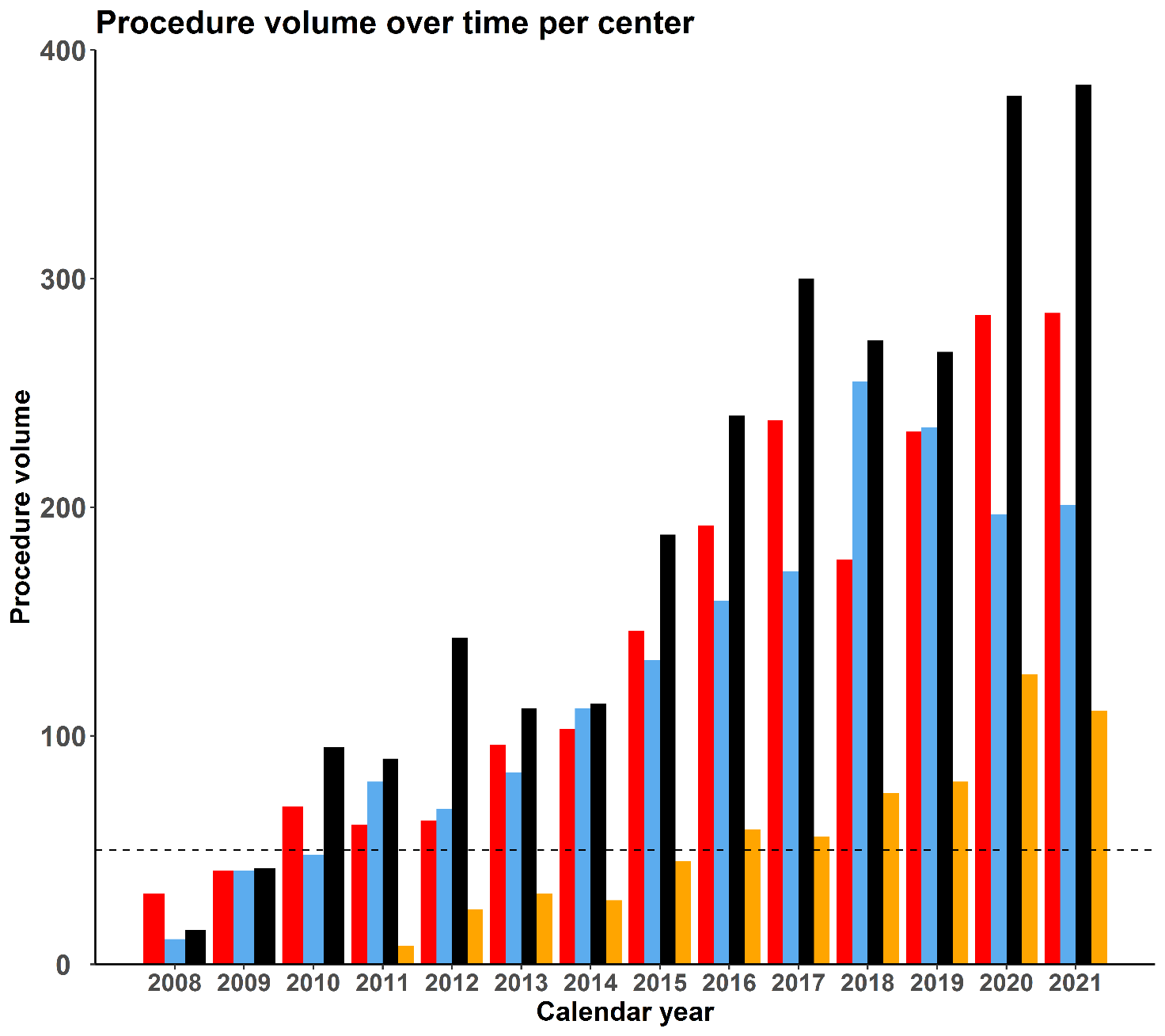


**Supplementary Figure 3**


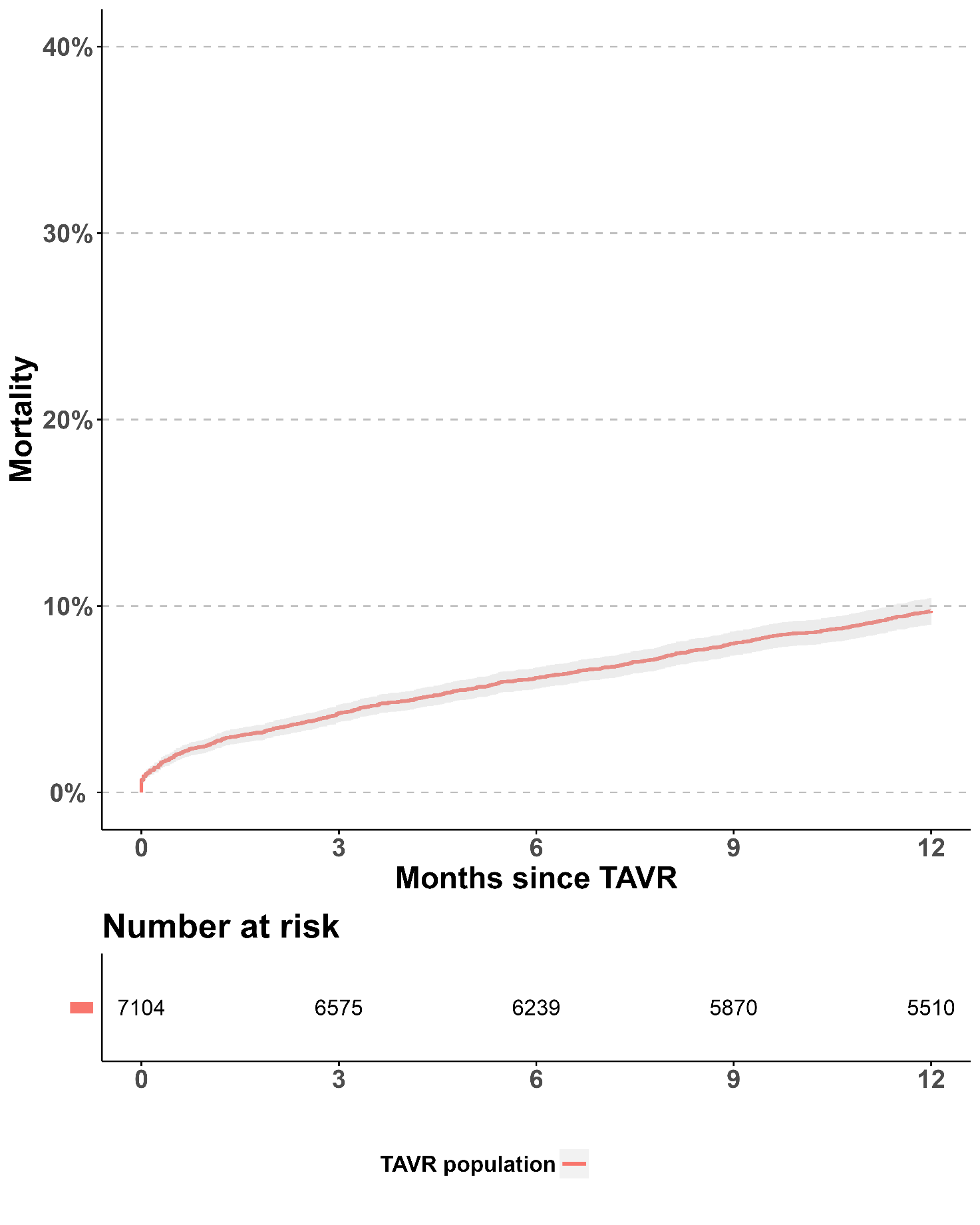


**Supplementary Figure 4**


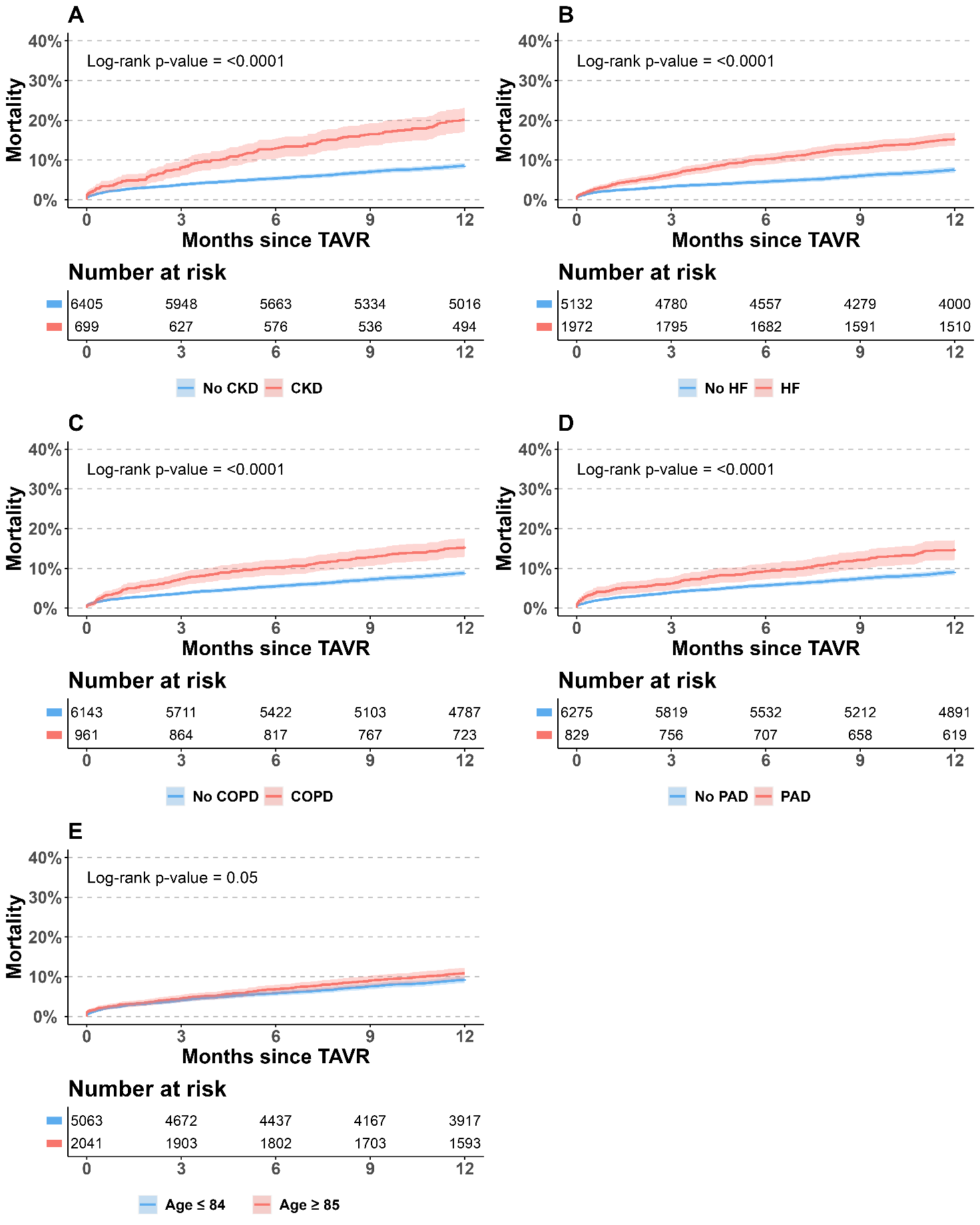


**Supplementary Figure 5**

**
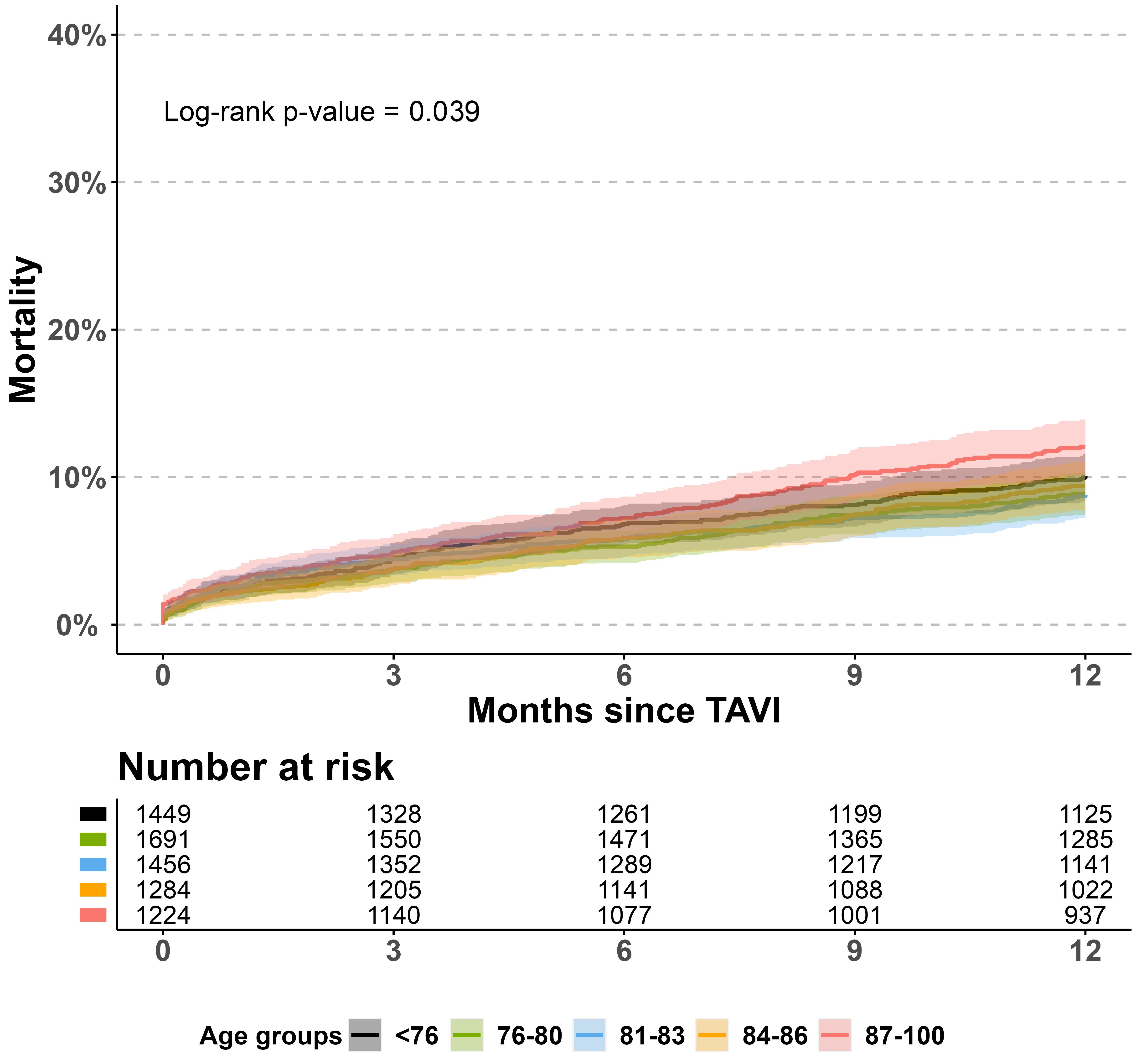
**

**Supplementary Figure 6**


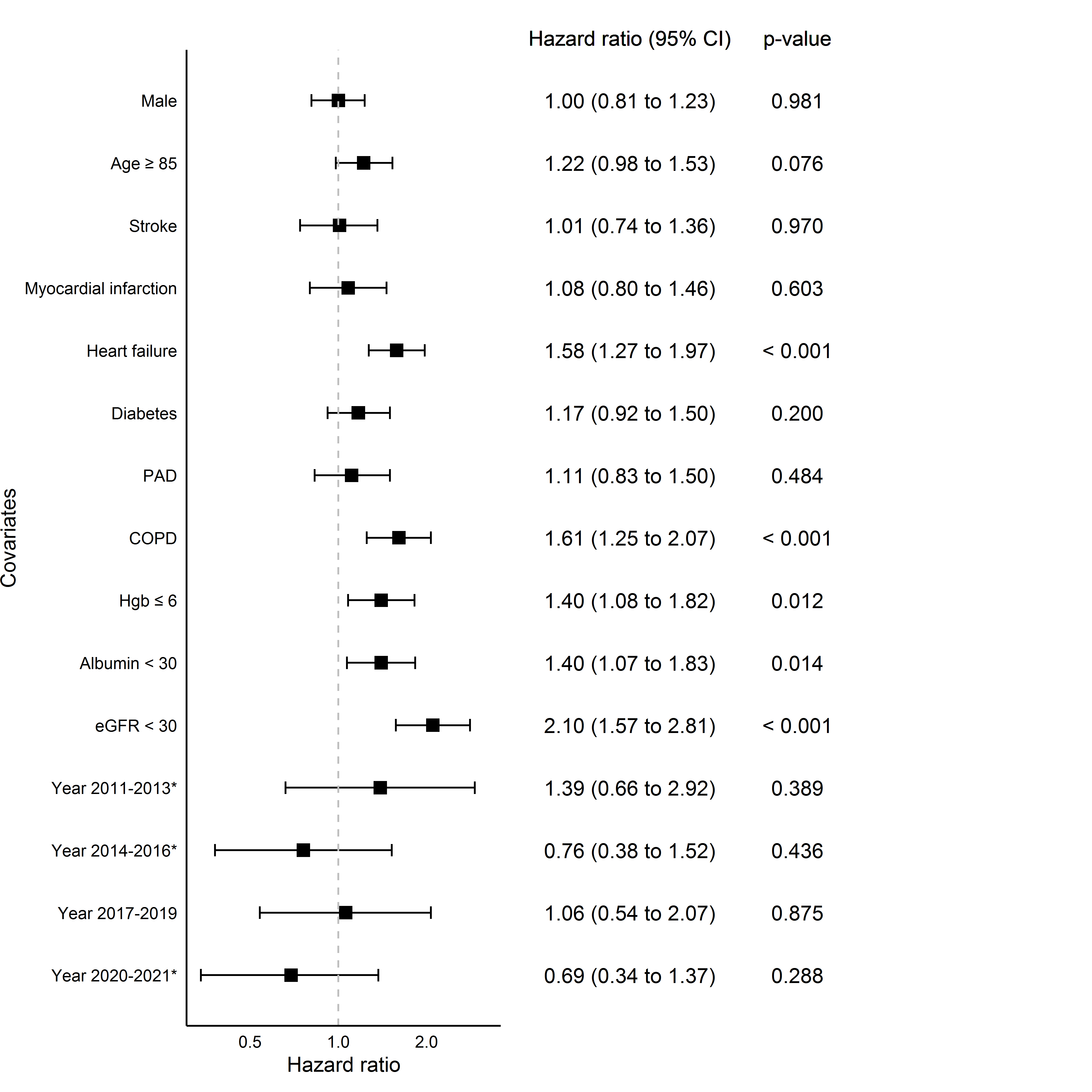


**Supplementary Figure 7**


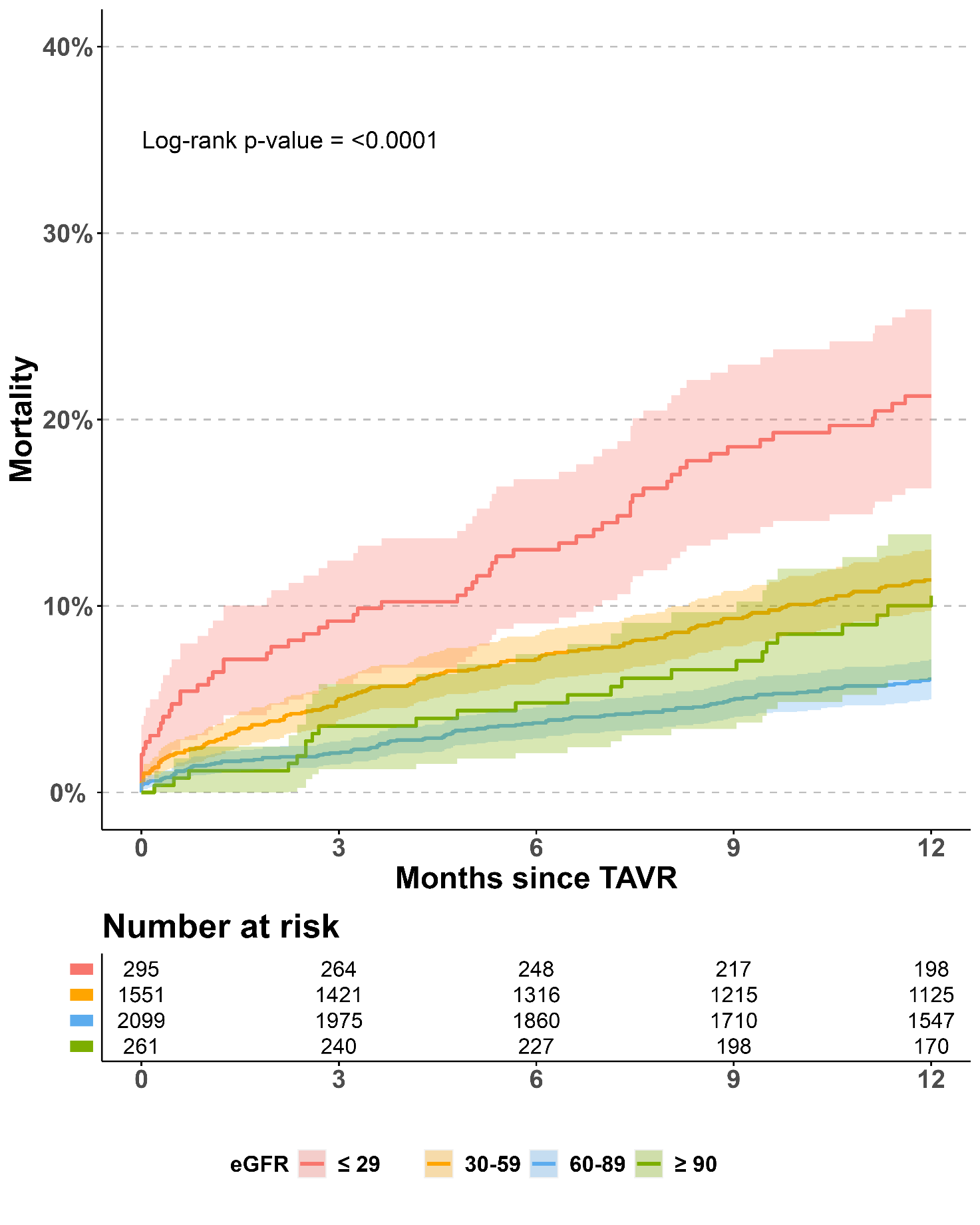

Supplement: Supplementary data 1 [file mmc1.docx]
